# Perception and control of allergic rhinitis in primary care

**DOI:** 10.1038/s41533-020-00195-8

**Published:** 2020-08-20

**Authors:** Pascal Demoly, Isabelle Bossé, Pascal Maigret

**Affiliations:** 1 Division of Allergy, Department of Pulmonology, Hôpital Arnaud de Villeneuve, University Hospital of Montpellier, Montpellier, France; 2 Sorbonne Université, UMR-S 1136 INSERM, IPLESP, EPAR Team, Paris, France; 3 Allergology Office, La Rochelle, France; 4 Menarini France, Rungis, France

**Keywords:** Respiratory tract diseases, Respiratory signs and symptoms

## Abstract

Perception of a chronic illness is a driver of patient behaviour that may impact treatment outcomes. The cross-sectional PETRA study was designed to describe the links between disease perception, patient behaviour and treatment outcomes in adults with allergic rhinitis (AR). Overall, 687 French general practitioners (GPs) included 1929 analysable patients (mean age: 39 years; intermittent/persistent symptoms: 46.2/52.3%). Of the patients, 14.1% had also been diagnosed with asthma; 71.7% had uncontrolled AR (ARCT score < 20), and 53.6% had a good perception of their illness (BIPQ score < 5). Factors significantly associated with poor perception of AR were ENT (ear/nose/throat) complications, nasal pruritus, uncontrolled AR and asthma. A strong negative correlation was observed between the BIPQ and ARCT scores: the poorer the patient’s perception, the less the AR was controlled. Although no causal relationship could be drawn, GP-driven improvement of AR perception could lead to better control of symptoms.

## Introduction

According to the World Health Organization (WHO), chronic diseases are the leading cause of morbidity and mortality worldwide, accounting for 43% of the global burden of disease (as per the 2002 report; 60% expected in 2020). Chronic diseases are mainly related to ageing of the population, lifestyle and environmental changes. Poor control of chronic diseases represents a public health burden and, consequently, patients need to be managed with the best evidence-based strategies possible, both at the patient and the community level^1^.

There is no consensual definition of ‘disease control’, but it could be described as the achievement of therapeutic objectives, or a reduction of symptom severity to acceptable levels through optimised treatment. Control of a chronic disease therefore requires that treatments be adjusted for individual comorbidities and risk factors, as per guidelines, as well as the patient’s personal involvement. Patient care is therefore moving from ‘bulk’ to stratified medicine, pending future personalised and precision medicine. The personal involvement of each patient is correlated with his/her perception of the disease and the associated treatments used for its control. Disease perception corresponds to cognitive and emotional representations of the illness and health threat, and encompasses several dimensions such as identity, consequences, cause, timeline, cure or control^2^. It is possible to activate a virtuous circle where perception and control can be improved, as shown with asthma^3,4^.

The prevalence of allergic rhinitis (AR) is high (around 400 million people worldwide^5^, nearly a third of the adults in France in 2009^6^), but it is often poorly self-recognised by patients, and also poorly controlled^7^. In 2001, at the initiative of the WHO, in the framework of the first ARIA (Allergic Rhinitis and its Impact on Asthma) workshop, a group of experts proposed a classification of AR in order to establish a consensual therapeutic approach based on scientific and clinical evidence. In the 2008 update of the ARIA guidelines, the principle that the upper and lower respiratory tracts are a continuum forming a unified airway was reaffirmed, and AR was reclassified based on clinical symptoms and quality of life scores^8^. Although AR is described by patients as disabling, care is neither optimal nor consistent with recommendations^9–11^ and, as shown in France, patients with severe AR consult a doctor on average 7 years after the onset of the disease^12^. The economic burden of the disease is weighed down by inadequate patient management^13^.

In this context, the PETRA study was designed to assess the management of AR by patients and their general practitioners (GP), and to describe the links between disease perception, patient behaviour and treatment outcomes. The main objective of the study was to precisely identify and describe factors associated with poor perception of the disease in a population of patients with AR.

## Results

### Characteristics of patients

Overall, 687 GPs participated in the PETRA study and a total of 2001 patients were included between May and October 2017. Of the patients, 1929 were retained for analysis as they met all the selection criteria and had returned their self-questionnaires. Most patients (88.7%) were included between May and July. The characteristics of the patients and their AR are presented in Table 1. Their medical care, including previous treatments and those prescribed on the visit day, are described in Table 2. Many patients (40.3%) declared they were not satisfied with their AR treatment. Regarding the patients’ knowledge of the disease, most of them (81.1%) knew that allergy was an immune system disorder; 60.5% cited asthma as a complication of AR and 73.6% thought that AR was a risk factor for developing asthma. In addition, 63.9% indicated that AR is a chronic and incurable disease. Almost all patients were convinced that prescription drugs were more effective than over-the-counter drugs (96.3%), and that limiting allergen exposure was an effective preventive measure (90.9%).

**Table 1:** Characteristics of patients and of allergic rhinitis.

Characteristics	Description	Analysed population *N* = 1929
Sex	Male/Female	49.8%/50.2%
Age		38.8 ± 14.4 years
	[18–30]/[30–50]/>50 years	34.8%/43.0%/22.2%
Living area	Big city/urban zone/village/rural area	12.1%/40.6%/34.0%/13.3%
Smoking	No/passive/active	73.4%/6.8%/19.8%
Familial history of allergy	Yes	53.6%
Seniority of AR	First episode	17.6%
	Duration from onset (if >1st episode)	10.2 ± 9.0 years
ARIA classification	Mild and intermittent	28.4%
	Moderate or severe and intermittent	18.5%
	Mild and persistent	14.1%
	Moderate or severe and persistent	39.0%
	Mild/moderate or severe	42.6%/57.4%
	Intermittent/persistent	46.9%/53.1%
PAREO questionnaire^a^	Total score	9.0 ± 2.7
Nasal itching	None or mild/moderate or severe	39.3%/60.7%
Anosmia	None or mild/moderate or severe	68.4%/31.5%
Rhinorrhea	None or mild/moderate or severe	18.0%/82.0%
Sneezing	None or mild/moderate or severe	17.2%/82.8%
Nasal obstruction	None or mild/moderate or severe	27.6%/72.5%
Ocular symptoms	Yes	64.1%
	Duration from onset (if yes)	6.6 ± 8.6 years
Comorbidities	At least one	30.4%
	Asthma	14.1%
	Atopic eczema	12.3%
	Contact dermatitis	8.2%
	Nasal polyps	4.5%
	Allergic keratitis	1.4%
History of ENT complications	At least one	20.2%
	Sinusitis	13.8%
	Recurrent ENT infections	7.6%
	Otitis media with effusion	2.6%
Aeroallergen responsible for AR	Known/unknown	60.3%/39.7%
	If known:	
	Grass pollen	74.4%
	Mites	44.9%
	Tree pollen	44.7%
	Herbaceous pollen	29.5%
	Animal dander	18.8%
	Fungal spores	8.9%
	Polysensitisation	66.9%
Other known allergens responsible for allergy	At least one	9.8%
	Food	4.5%
	Chemical	5.5%

^a^Each symptom scored 0—absent, 1—mild, 2—moderate, or 3—severe, for a total score ranging from 0 to 15. Values are mean ± standard deviation for continuous variables, and % of classes for categorical variables.

**Table 2:** Medical care for allergic rhinitis.

Characteristics		Analysed population *N* = 1929
AR follow-up	First consultation for AR	34.3%
	Regular follow-up by GP	57.4%
	Regular follow-up by an allergy specialist	2.0%
	At least one consultation with an allergy specialist	36.9%
	Unknown care course	6.2%
Main consultation reason	Immediate release	56.6%
	Treatment renewal	35.1%
	Prescription of long-term treatment	30.7%
	Change/adjustment of ongoing treatment	16.4%
Ongoing symptomatic treatment	At least one	64.6%
	Oral anti-H1	59.9%
	Intranasal steroids	27.6%
	Intranasal anti-H1	14.1%
	Intraocular cromone	11.0%
	Intraocular anti-H1	10.2%
	1/2/3/>3 therapies	19.6%/22.5%/17.8%/4.6%
	Self-medication	15.7%
Prescribed symptomatic treatment	At least one	99.5%
	Oral anti-H1	97.7%
	Intranasal steroids	47.8%
	Intranasal anti-H1	24.7%
	Intraocular cromone	21.7%
	Intraocular anti-H1	19.5%
	1/2/3/>3 therapies	20.1%/34.2%/35.0%/10.7%
Allergen immunotherapy	Yes, whenever	7.4%
	Yes, ongoing	2.2%
	Against:	
	Mites	5.9%
	Grass pollen	5.7%
	Tree pollens	2.7%
	Herbaceous pollen	1.8%
	Animal dander	1.6%
	Fungal spores	0.9%
	Other	0.4%
Referred to a specialist	Yes:	15.1%
	Allergy specialist	12.2%
	Lung specialist	2.8%
	ENT specialist	2.6%
	Dermatologist	0.7%
Sick leave prescription	Yes	1.6%
	Duration (if yes)	4.8 ± 2.7 days

Values are mean ± standard deviation for continuous variables, and % of classes for categorical variables.

The mean Allergic Rhinitis Control Test (ARCT) score was 17.3 ± 3.5 points, with AR considered as uncontrolled in 71.7% of patients (ARCT score < 20).

### Factors associated with poor disease perception

**Fig. 1:**
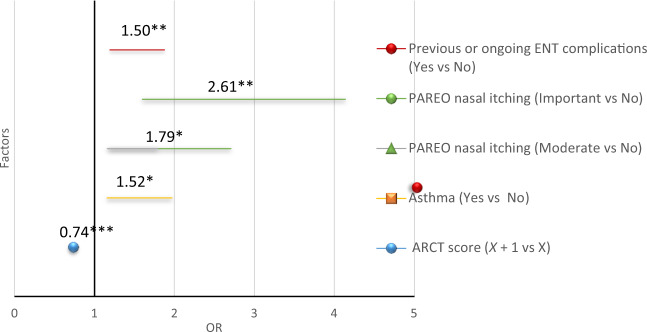
Factors associated with poor perception of AR. Poor perception of AR was defined by a BIPQ score [5–7]. Odds ratios (OR) were determined using multivariate logistic regression. An OR > 1 indicates an excess risk of poor perception; the higher the ARCT score, the better the control. The bar represents the 95% confidence interval (95%CI) of OR *p < 0.05; **p < 0.001; ***p < 0.0001 (Wald test).

The mean Brief Illness Perception questionnaire (BIPQ) score was 4.8 ± 1.0 points, with perception of AR considered as good in 53.6% of patients (BIPQ score [0–5]), poor in 44.6% (BIPQ score [5–7]) and very poor in 1.8% (BIPQ score [7–10]). Univariate analysis identified several factors significantly associated with poor disease perception: smoking, intense anosmia, ocular symptoms, AR comorbidities (asthma, atopic eczema, nasal polyps, allergic keratitis, contact dermatitis, ear-nose-throat [ENT] complications) and poor control of AR. Subsequent multivariate logistic regression analysis demonstrated the links between the factors significantly associated with poor disease perception, i.e. BIPQ score [5–7] (Fig. 1): the presence of ENT complications (OR: 1.5; 95%CI: [1.2; 1.9]), significant or moderate nasal pruritus (OR: 2.6; 95%CI: [1.6; 4.1] and 1.8; 95%CI: [1.2; 2.7], respectively), uncontrolled AR (OR: 0.7 for 1 point more on the ARCT score; 95%CI: [0.7; 0.8]) and asthma (OR: 1.5; 95%CI: [1.2; 2.0]). The factors significantly associated with a very poor perception of AR (BIPQ > 7) were asthma (OR: 5.1; 95%CI: [2.5; 10.4]), allergic keratitis (OR: 9.6; 95%CI: [2.5; 36.9]) and uncontrolled AR (OR: 0.5 for 1 point more on the ARCT score; 95%CI: [0.5; 0.6]).

**Fig. 2:**
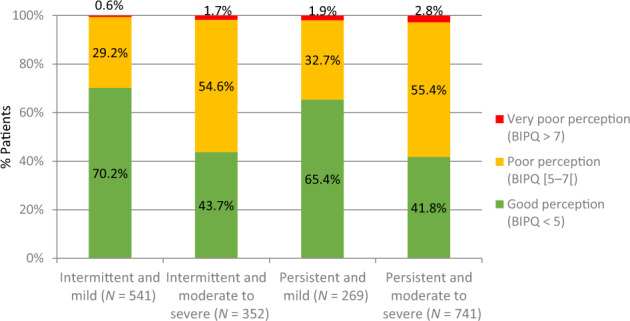
% patients with various perception (BIPQ) scores in the 4 ARIA severity groups. Perception of AR according to the ARIA classification.

Patients with mild AR symptoms had a better perception of their disease (according to the BIPQ score) than those with moderate to severe symptoms, as shown in Fig. 2.

### Correlations between disease perception and disease control

A strong negative correlation was observed between the BIPQ and ARCT scores (*R* = −0.57; *p* < 0.0001): the poorer the patient’s perception, the less the AR was controlled. The correlation was mainly based on a few specific questions of the BIPQ, such as ‘How does your disease affect your life?’ (*R* = −0.59; *p* < 0.0001), ‘How does your disease affect you emotionally?’ (*R* = −0.50; *p* < 0.0001) and ‘What is the frequency of your symptoms?’ (*R* = −0.45; *p* < 0.0001). Among the well-controlled patients (ARCT > 20), 84.9% had a good perception of their illness (BIPQ < 5) versus 41.1% of the poorly controlled patients (ARCT ≤ 20; *p* < 0.001). The mean BIPQ score was also significantly lower in well-controlled patients (indicating a better perception) than in others (4.0 versus 5.1; *p* < 0.001).

### Factors associated with poor disease control

Among the patients with poorly controlled AR, 10.1% thought that reducing exposure to allergens was not an effective preventive measure, whereas that opinion was shared by only 5.9% of the patients in whom the disease was well controlled. Almost all the patients believed that prescription treatments were more effective than over-the-counter medications. However, 4.2% of the poorly controlled patients believed that prescription treatments were not more effective compared to 2.5% of the well-controlled patients. Allergen immunotherapy was perceived equally by all patients regardless of the level of AR control with 53.7% declaring that it could cure AR.

## Discussion

PETRA was a large cohort of adults with AR (1929 patients analysed) observed in real-life conditions in a primary care setting (GP’s office) very representative of the management of AR patients in France. Almost 90% of the patients were recruited into the cross-sectional study between May and July (2017), which is a high-risk period for many aeroallergens. Pollens, which are abundant in this period, were often the causal agents of AR in this cohort. Symptom onset likely prompted the visit to the GP for most patients, particularly for those in whom the causative allergen remained unidentified or for whom the visit was a first for AR (about one third of the cohort in each situation).

The study data confirmed that disease control of patients managed in a primary care setting was poor (71.7% of patients), which is consistent with the results of another French cohort followed by GPs^14^. In addition to the ARCT questionnaire assessing disease control, we also applied the BIPQ questionnaire to assess disease perception. The main objectives of the PETRA study were to identify the factors associated with poor perception of AR and how perception relates to control. Interestingly, the ARIA classification (four classes) was not found to be significantly associated with AR perception, although, in addition to annoying symptoms (nasal pruritus) and comorbidities (asthma, keratitis, ENT complications), a low ARCT score was significantly associated with a high BIPQ score: the poorer the patient’s perception, the less the AR was controlled. This observation, derived from the multivariate analysis, was confirmed by the significant negative correlation found between the ARCT and BIPQ scores. It is now recognised that disease perception is linked to patient behaviour. Several studies conducted worldwide in various clinical situations all suggest that interventions designed to change disease perception can improve health incomes^4,15^. In this context, initiating a virtuous circle in which disease control is improved together with disease perception and vice versa can only be beneficial, and disease information is key to this. First, a public health campaign, which should also involve pharmacists, could encourage patients to see a GP for any recurrent episodes of runny nose, sneezing and eye-watering. A third of the patients in the PETRA cohort had seen their GPs for the first time for AR symptoms, and only half the patients were followed regularly for the condition despite their symptoms being present for an average of 8 years (a delay consistent with a previous French survey^16^) with the treatment appearing inadequate in most cases (low use of intranasal steroids or allergen immunotherapy, for instance). Secondly, once the GP has been approached by the patient, he/she becomes the front-line intermediary regarding the dispensing of information. Most patients recognise AR symptoms fairly well and have a good knowledge of the allergenic agent responsible for their condition as shown in this study; some of them, however, do not link AR to its potential complications and comorbidities. Efforts should be made to inform patients about the causes and consequences of AR, its frequent association with asthma, and the need to monitor and care for the entire respiratory system, the aim being to increase disease awareness and AR control. Asthma and keratitis are warning signs that should be used to raise patients’ awareness about their disease as they are significantly associated with very poor perception of AR. Therapeutic education is also crucial in disease management. The study showed that about 40% of patients were dissatisfied with their treatment, although it had been prescribed by a physician in most cases: this result seems to indicate that overall management is poorly adapted. In addition, about one third of patients were unable to indicate that AR is a chronic disease. This suggests that most patients are not aware that AR requires long-term management in addition to short-term treatment during periods of exacerbation. As for any chronic disease, GPs should encourage patients to become active participants of their own care. Regular patient follow-up should not be considered as an extraneous expense as the visits can be used to reinforce education and compliance and avoid disease exacerbations, which certainly generate the most costs in any chronic disease, whether psychologically, functionally or socially, and sometimes even alter the prognosis of the disease in the long term. Follow-up visits can also serve to update patients on any new knowledge gained about the disease^13^.

General practitioners also play a crucial role in referring AR patients to allergy specialists when necessary to step up treatment and avoid disease worsening; decision trees can help GPs to determine when referral to a specialist might help^17^. In this study, 15% of patients were referred to an allergy specialist at the end of the visit. Patients followed by GPs present milder clinical profiles than those followed by specialists. Indeed, 56.6% of patients in the PETRA cohort had moderate to severe symptoms, and the pattern was persistent in 52.3% of them, whereas the rates were 80% and 65.8%, respectively, in the French REALIS study conducted by lung or allergy specialists. In addition, the prevalence of asthma was 14.1% in the PETRA study versus 40.3% in the REALIS study^18^. In this cohort, only 2% of patients regularly saw an allergy specialist. Although the subgroup is very small, these patients tended to have a better knowledge of their disease and were treated more frequently with allergen immunotherapy.

Defending the right of access of patients with respiratory allergies to the best possible care is a public health matter that requires GPs and allergy specialists to combine their efforts. Medicine is at best stratified nowadays and hopefully it will soon become personalised thanks to such optimised patient care. Nonetheless, access to allergists in France is difficult due to their rarity. This may change in the future, however, with allergology having become a full specialty in 2017 and the increase in awareness of public authorities, health professionals and the general public about the consequences of a disease that is still too often trivialised or ignored^16^.

This study presents some limitations. First, the sample of GPs may not be representative of French practitioners (excess of men, non-homogenous regional repartition, participation likely to be related to a specific interest in allergy, etc.). Secondly, the cross-sectional study provides a single picture of a cohort of AR patients and only suggests ways to improve overall disease control, which remain to be explored.

In conclusion, the PETRA cohort included a high proportion of patients with moderate to severe AR symptoms and a low level of disease control. It appeared that many patients were not satisfied with their treatment and did not perceive their disease very well. Poor disease perception was associated with the presence of ENT complications, moderate to severe nasal pruritus, asthma and poor disease control. Although no causal relationship could be drawn from this cross-sectional study, results suggest that enhancing perception of AR could be beneficial to patients and lead to better control of symptoms. GPs, as front-line health professionals with regard to patients, are key to improving their cognitive representations of AR.

## Methods

### Design and regulatory context

PETRA was an observational, cross-sectional, prospective, multicentre study conducted in France by GPs. The protocol, patient information sheet and all other documents were submitted to and approved by the Advisory committee on information processing in health research matters (*Comité Consultatif sur le Traitement de l’Information en Matière de Recherche dans le Domaine de la Santé*) and the National commission on data processing and liberties (*Commission Nationale de l’Informatique et des Libertés*) before the study started, in compliance with French legislation and ethical regulations. The study was not registered, as it was not mandatory in France for non-interventional studies at the time it was designed. Patients aged 18 years or more, already diagnosed with AR, or strongly presumed to be suffering from AR, were included during a routine visit after being informed about the study and having expressed their non-opposition to personal data collection as per currently applicable French regulations (written consent is not required for non-interventional studies).

### Data collection

General practitioners collected data on paper-based case report forms: socio-demographic characteristics, living conditions, history of AR including the ARIA classification of severity^8,19^ and the PAREO (*Prurit/*nasal pruritus*, Anosmie/*anosmia*, Rhinorrhée/*rhinorrhea*, Eternuements/*sneezing*, Obstruction nasale/*nasal obstruction) score for symptom intensity (each of the five symptoms graded from 0 to 3, for a total ranging from 0 to 15)^20^, diagnosis of AR, and ongoing and prescribed treatments. Patients filled in self-questionnaires about their knowledge of AR and associated diseases and treatments, disease control (ARCT questionnaire, the total score of which ranged from 5—poorest control to 25—best control^14^), and illness perception (BIPQ, the total scores of which ranged from 0—best perception to 10—poorest perception^2,15^).

### Statistical methods

The main objective of the study was to identify the factors associated with a poor perception of the disease, defined by a BIPQ score ≥ 5. Univariate tests were first used on predictive variables (chi^2^ or exact Fisher test for categorical variables, Student’s *t* test or non-parametric Mann−Whitney or Kruskal−Wallis tests for continuous variables), and were then followed by a step-by-step multivariate logistic regression analysis to determine the odds ratios (OR) and their 95% confidence intervals (95%CI) and *p* values. The BIPQ scores and sub-scores were described taking into account the ARIA classification (mild and intermittent/mild and persistent/moderate to severe and intermittent/moderate to severe and persistent), and the degree of disease control (ARCT score ≥ 20/<20). The Pearson correlation coefficient was calculated between the BIPQ and ARCT scores. There was no replacement of missing data for the explicative variable (BIPQ score). Sample size was calculated for 80% power and an alpha risk of 5% to allow identification of factors associated with a BIPQ class with an OR ≥ 1.5, assuming disease perception would be poor (BIPQ ≥ 5) in 50% of subjects and the smallest class of associated factors at 9%. On this basis, according to the formula of Casagrande et al.^21^, 2362 analysable cases were required for the study. Considering a 5% rate of unanalysable data, it was planned to include 2486 patients in the survey.

### Reporting summary

Further information on research design is available in the Nature Research Reporting Summary linked to this article

## Supplementary information

Reporting Summary

## Data Availability

The data that support the findings of this study are available from the corresponding author upon reasonable request.
