# Supplementary material for: Perception and control of allergic rhinitis in primary care
Source: NPJ Prim Care Respir Med. 2020 Aug 20;30:37. doi: 10.1038/s41533-020-00195-8 (PMC7441396; doi:10.1038/s41533-020-00195-8)
Supplement: Supplementary file 1 — Reporting Summary [file 41533_2020_195_MOESM1_ESM.pdf]

nature research

Corresponding author(s): *Double-blind peer review submissions: write DBPR and your manuscript number here instead of author names.*

Last updated by author(s): *YYYY-MM-DD*

Reporting Summary

Nature Research wishes to improve the reproducibility of the work that we publish. This form provides structure for consistency and transparency in reporting. For further information on Nature Research policies, see our Editorial Policies and the Editorial Policy Checklist.

Statistics

For all statistical analyses, confirm that the following items are present in the figure legend, table legend, main text, or Methods section.

n/a	Confirmed	
☐	☒	The exact sample size (*n*) for each experimental group/condition, given as a discrete number and unit of measurement
☒	☐	A statement on whether measurements were taken from distinct samples or whether the same sample was measured repeatedly
☐	☒	The statistical test(s) used AND whether they are one- or two-sided<br>*Only common tests should be described solely by name; describe more complex techniques in the Methods section.*
☒	☐	A description of all covariates tested
☒	☐	A description of any assumptions or corrections, such as tests of normality and adjustment for multiple comparisons
☐	☒	A full description of the statistical parameters including central tendency (e.g. means) or other basic estimates (e.g. regression coefficient) AND variation (e.g. standard deviation) or associated estimates of uncertainty (e.g. confidence intervals)
☒	☐	For null hypothesis testing, the test statistic (e.g. *F*, *t*, *r*) with confidence intervals, effect sizes, degrees of freedom and *P* value noted<br>*Give P values as exact values whenever suitable.*
☒	☐	For Bayesian analysis, information on the choice of priors and Markov chain Monte Carlo settings
☒	☐	For hierarchical and complex designs, identification of the appropriate level for tests and full reporting of outcomes
☒	☐	Estimates of effect sizes (e.g. Cohen's *d*, Pearson's *r*), indicating how they were calculated

Our web collection on statistics for biologists contains articles on many of the points above.

Software and code

Policy information about availability of computer code

Data collection	Paper CRFs and self-questionnaires, entered in an electronic database designed with Ennov Clinical.
Data analysis	SAS 9.4

For manuscripts utilizing custom algorithms or software that are central to the research but not yet described in published literature, software must be made available to editors and reviewers. We strongly encourage code deposition in a community repository (e.g. GitHub). See the Nature Research guidelines for submitting code & software for further information.

Data

Policy information about availability of data

All manuscripts must include a data availability statement. This statement should provide the following information, where applicable:

- Accession codes, unique identifiers, or web links for publicly available datasets
- A list of figures that have associated raw data
- A description of any restrictions on data availability

The data that support the findings of this study are available from the corresponding author upon reasonable request

Field-specific reporting

Please select the one below that is the best fit for your research. If you are not sure, read the appropriate sections before making your selection.

☒ Life sciences ☐ Behavioural & social sciences ☐ Ecological, evolutionary & environmental sciences

For a reference copy of the document with all sections, see nature.com/documents/nr-reporting-summary-flat.pdf

Life sciences study design

All studies must disclose on these points even when the disclosure is negative.

Sample size	Sample size was calculated for 80% power and an alpha risk of 5% to allow identification of factors associated with a BIPQ class with an OR ≥ 1.5, assuming disease perception would be poor (BIPQ ≥ 5) in 50% of subjects and the smallest class of associated factors at 9%. On this basis, according to the formula of Casagrande et al., 2362 analysable cases were required for the study. Considering a 5% rate of unanalysable data, it was planned to include 2486 patients in the survey
Data exclusions	1929 patients, out of 2001 included patients (96.4%), were retained for analysis as they met all the selection criteria and had returned their self-questionnaires
Replication	NA
Randomization	NA
Blinding	NA

Reporting for specific materials, systems and methods

We require information from authors about some types of materials, experimental systems and methods used in many studies. Here, indicate whether each material, system or method listed is relevant to your study. If you are not sure if a list item applies to your research, read the appropriate section before selecting a response.

Materials & experimental systems

n/a	Involved in the study	
☒	☐	Antibodies
☒	☐	Eukaryotic cell lines
☒	☐	Palaeontology and archaeology
☒	☐	Animals and other organisms
☐	☒	Human research participants
☐	☒	Clinical data
☒	☐	Dual use research of concern

Methods

n/a	Involved in the study	
☒	☐	ChIP-seq
☒	☐	Flow cytometry
☒	☐	MRI-based neuroimaging

Human research participants

Policy information about studies involving human research participants

Population characteristics	Adults with allergic rhinitis (AR); 49.8% male; mean age: 38.8 years (cf Table 1 for details)
Recruitment	Patients aged 18 years or more, already diagnosed with AR, or strongly presumed to be suffering from AR, were included during a routine visit to their general practitioner after being informed about the study and having expressed their non-opposition to personal data collection
Ethics oversight	The protocol, patient information sheet and all other documents were submitted to and approved by the Advisory committee on information processing in health research matters (Comité Consultatif sur le Traitement de l'Information en Matière de Recherche dans le Domaine de la Santé) and the National commission on data processing and liberties (Commission Nationale de l'Informatique et des Libertés) before the study started, in compliance with French legislation

Note that full information on the approval of the study protocol must also be provided in the manuscript.

Clinical data

Policy information about clinical studies

All manuscripts should comply with the ICMJE guidelines for publication of clinical research and a completed CONSORT checklist must be included with all submissions.

Clinical trial registration	The study was not registered, as it was not mandatory in France for non interventional studies at the time it was designed
Study protocol	Submitted as supplemental file
Data collection	Cross-sectional data collection (during a unique visit). Visits performed between May 2017 and October 2017
Outcomes	Primary outcome (patient reported): BIPQ<br>Secondary outcomes :<br>- Doctor reported: ARIA classification, PAREO score...<br>- Patient reported: ARCT questionnaire, disease knowledge...
